# Intermittent Hypoxia Mediates Caveolae Disassembly That Parallels Insulin Resistance Development

**DOI:** 10.3389/fphys.2020.565486

**Published:** 2020-11-26

**Authors:** Maider Varela-Guruceaga, Elise Belaidi, Lucie Lebeau, Ella Aka, Ramaroson Andriantsitohaina, Sophie Giorgetti-Peraldi, Claire Arnaud, Soazig Le Lay

**Affiliations:** ^1^INSERM UMR1063, Oxidative Stress and Metabolic Pathologies, University of Angers, SFR ICAT, Angers, France; ^2^Univ. Grenoble Alpes, Inserm, CHU Grenoble Alpes, HP2, Grenoble, France; ^3^Université Cote d’Azur, Inserm, C3M, Team Cellular and Molecular Physiopathology of Obesity, Nice, France

**Keywords:** OSA (obstructive sleep apnea), intermittent hypoxia (IH), insulin resistance, caveolin, cavin, caveolae, adipocyte

## Abstract

Repetitive complete or incomplete pharyngeal collapses are leading to chronic intermittent hypoxia (CIH), a hallmark feature of obstructive sleep apnea (OSA) syndrome responsible for many metabolic disorders. In humans, an association between OSA and insulin resistance has been found independently of the degree of obesity. Based on our previous work showing that hypoxia applied to adipocytes led to cellular insulin resistance associated with caveolae flattening, we have investigated the effects of CIH on caveolae structuration in adipose tissue. Original exploratory experiences demonstrate that 6 weeks-exposure of lean mice to CIH is characterized by systemic insulin resistance and translates into adipocyte insulin signaling alterations. Chronic intermittent hypoxia also induces caveolae disassembly in white adipose tissue (WAT) illustrated by reduced plasma membrane caveolae density and enlarged caveolae width, concomitantly to WAT insulin resistance state. We show that CIH downregulates caveolar gene and protein expressions, including cavin-1, cavin-2, and EHD2, underlying molecular mechanisms responsible for such caveolae flattening. Altogether, we provide evidences for adipose tissue caveolae disassembly following CIH exposure, likely linked to cavin protein downregulation. This event may constitute the molecular basis of insulin resistance development in OSA patients.

## Introduction

Obstructive sleep apnea (OSA) is characterized by frequent episodes of partial or complete upper airway obstructions during sleep leading to repetitive apneas and hypopneas (Levy et al., 2015). Obstructive sleep apnea constitutes a major health problem due to its high prevalence and its close association with the obesity epidemic. Repetitive airway pharyngeal collapses lead to chronic intermittent hypoxia (CIH), a hallmark feature of OSA responsible for metabolic disorders including cardiovascular co-morbidities, metabolic syndrome and type 2 diabetes (Levy et al., 2015). Clinical studies have established an independent association between OSA and insulin resistance, independently of the degree of obesity (Ip et al., 2002; Fredheim et al., 2011; Murphy et al., 2017). Mimicking OSA in rodents by exposing animals to repetitive hypoxia/reoxygenation cycles confirms the causal link between CIH, systemic insulin resistance and insulin signaling alterations in white adipose tissue (WAT), independently of obesity (Murphy et al., 2017; Thomas et al., 2017). Possible mechanisms underlying insulin resistance development includes CIH-induced HIF-1 (hypoxia inducible factor-1) activation, the master regulator of oxygen homeostasis (Belaidi et al., 2016; Khalyfa et al., 2017).

Obesity is another pathophysiological situation associated with WAT hypoxia consecutive to a maladaptive vascularization with regard to adipocyte expansion (Trayhurn, 2014). White adipose tissue limited oxygen supply also triggers chronic low-grade inflammation and increases fibrosis, cellular senescence and adipocyte death, which overall constitute major trigger of obesity-associated metabolic complications, including type 2 diabetes (Crewe et al., 2017). In cultured adipocytes, hypoxia inhibits insulin-induced pathways and induces insulin resistance through an HIF-1-dependent mechanism (Regazzetti et al., 2009). Accordingly, specific overexpression of HIF-1 in adipocytes leads to the development of insulin resistance in mice (Halberg et al., 2009). We have moreover demonstrated that, in adipocytes, hypoxia leads to membranous caveolae disappearance, by downregulating the expression of caveolae protein adaptors, the cavins, through an HIF-dependent mechanism (Regazzetti et al., 2015). Finally, we have identified a strong positive correlation between adipose cavin-2 downregulation and the HOMA-IR (homeostasis model assessment of insulin resistance) of obese diabetic patient (Regazzetti et al., 2015) highlighting the importance of functional plasma membrane caveolae in insulin signaling.

Caveolae are specialized membrane microdomains, which function as signaling platforms and participate in several cellular processes (reviewed in Parton et al., 2020). The main structural proteins that regulate the structure and the function of caveolae are the caveolins and cavins. Caveolae are present in most cell types, but are remarkably enriched in adipocytes, where they are estimated to cover ∼30–50% of the adipocyte cell surface, and play an important role in insulin signaling and in fat storage as illustrated by lipoatrophic and insulin resistance phenotypes of caveolae-deficient animal models (Cohen et al., 2003). Indeed, many insulin signaling mediators are localized in caveolae and caveolin-1 (Cav-1) scaffolding domain interacts and positively regulates the insulin receptor. It has been shown by several groups including ours that downregulation of cavins leads to caveolae flattening demonstrating the importance of cavin proteins for the membrane caveolae structure (Briand et al., 2014; Parton et al., 2020). Therefore, caveolae disassembly appears as a critical adipocyte membrane remodeling process, which can be impacted by hypoxia, and thereby could participate in the development of insulin resistance in WAT.

The impact of CIH on adipose caveolae structure has never been explored. Therefore, the aim of our study is to investigate the effects of CIH on caveolae structure in WAT in a mouse model of CIH-induced insulin resistance.

## Materials and Methods

All reagents, unless otherwise specified, were obtained from Sigma Aldrich (Saint-Quentin Fallavier, France).

### Animal Experimentations and Experimental Design

Animal studies were conducted according to the French guidelines for the care and use of experimental animals (agreement number APAFIS#8840-2017020613274765).

**FIGURE 1:**
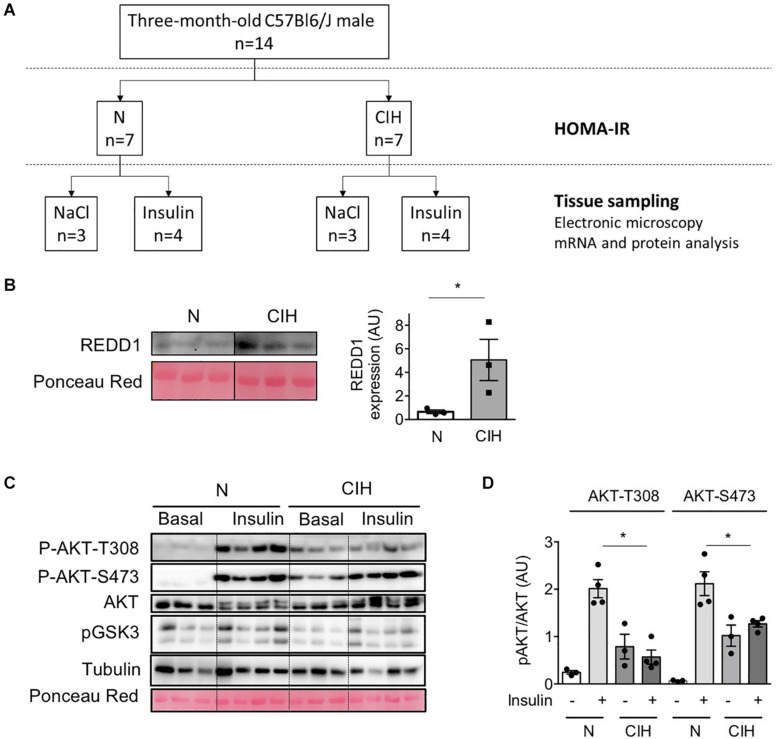
Long-term exposure to CIH, hypoxia-related protein expression and alterations of insulin signaling in adipose tissue. **(A)** Experimental Design: Fourteen 3-months-old C57Bl6/J male mice were randomly submitted to 6 weeks exposure to chronic intermittent hypoxia (CIH, *n* = 7) or normoxia (N, *n* = 7). After 6 weeks CIH and 6 hours fasting, mice were injected with insulin (0.5 U/kg total body weight, i.p, *n* = 4 per group) or NaCl (100 μL/10g body weight, i.p, *n* = 3 per group) and euthanatized 15 min later. **(B)** Increased REDD1 expression following CIH traduces HIF-1 transactivation. Western Blot of HIF-1 target protein REDD1 was performed in epididymal WAT lysates collected from animals exposed to normoxia (N) or CIH for 6 weeks. The line separates normoxic (N) and epididymal WAT samples from mice exposed to 6 weeks CIH, run on the same blot, but not loaded adjacently. Data are mean ± sem (*n* = 3 per group). *p < 0.05 vs. normoxic mice (following Student *t*-test). **(C,D)** CIH alters insulin signaling in adipose tissue. AKT phosphorylation (on T-308 and S-473) was investigated in epididymal WAT lysate samples 15 min after NaCl (basal) or insulin injection in 6 h-fasted mice previously exposed to normoxia (N) or CIH for 6 weeks. The phosphorylated-to-total ratios were calculated and is presented for N and CIH **(C)**. Data are mean ± sem (*n* = 3 in basal and 4 in insulin condition, in each group). *p < 0.05 vs. normoxic mice (following one-way ANOVA test, corrected by Tukey’s multiple comparison test).

Exploratory experiments were conducted using fourteen 3-month-old C57Bl6/J male mice that were randomly submitted to 6-weeks exposure to CIH (*n* = 7) (5% inspired oxygen fraction (FiO_2_) for 30 s and 21% FiO_2_ for 30 s, 8 h/day during sleeping time) or normoxia (N, *n* = 7) (similar air flow without modification of oxygen), as previously described (Murphy et al., 2017; Thomas et al., 2017); (see experimental design, Figure 1A). The week before sacrifice, all mice were fasted for 6 h and blood samples were drawn from the tail to determine fasting glycemia using a glucometer (One Touch VERIO) and insulinemia following manufacturer’s instructions (ELISA kit, Millipore). The HOMA-IR adjusted to rodents was calculated as ([glucose (mg/dL)/18] × [insulin (ng/mL)/0.0347])/108.24. HOMA-IR is used to estimate the insulin sensitivity from fasting plasma insulin and glucose concentration, and insulin resistance is characterized by increased HOMA-IR, compared to physiological condition. Finally, after 6 weeks CIH and 6 h fasting, mice were weighted, injected with insulin (0.5 U/kg total body weight, i.p, *n* = 4 per group) or NaCl (100 μL/10 g body weight, i.p, *n* = 3 per group) and euthanatized 15 min later by cervical dislocation, as previously described (Murphy et al., 2017; Thomas et al., 2017), Figure 1A. Tissues (epididymal and subcutaneous WAT and liver) were weighted, flash frozen in liquid nitrogen and stored at −80°C until use or immediately fixed for electron microscopy processing. Epididymal WAT were used for biochemical and histological analysis.

### Real-Time Quantitative RT-PCR

Epididymal WAT was used for real-time quantitative PCR (RT-qPCR) as previously described (Briand et al., 2014). mRNA expression was normalized to GAPDH expression. Primer sequences are available on request.

### Western-Blotting

Epididymal WAT was homogenized by mechanical homogeneization (Ultra-Turrax IKA T10 or Precellys tissue homogenizer) in ice-cold buffer as previously described (Briand et al., 2014; Dumas et al., 2020). Protein concentrations were determined using the Quick Start Bradford assay (Bio-Rad), and samples were subjected to SDS-PAGE and blotted according to standard procedures. Appropriate IRDye secondary antibodies were used for protein detection with Odyssey imaging system (LI-COR Biosciences) or HRP linked antibodies were used and immunoblots were revealed using PXi4 GeneSys imaging system. Antibodies used for IRdye imaging system were: b-actin (Sigma Aldrich, A5316), aP2 (Santa Cruz, SC271529), Cav-1 (BD Biosciences, 610407), cavin-1 (Merck Millipore, ABT131), cavin-2 (R&D Systems, AF5759), cavin-3 (Merck Millipore, ABE1043). Antibodies used for HRP revelation were: Akt-pT308 (Cell signaling Technology, CST13038), Akt-pS473 (CST4058), Akt(pan) (CST9272), REDD1 (Proteintech, 10638-1-AP), Tubulin (Sigma-Aldrich, T6199). Immunoblot quantifications were realized using Image Studio^®^ or Fiji softwares (Schindelin et al., 2012). Actin and aP2 proteins are used as loading controls. Entire crude blots are presented as Supplementary Material.

### Electron Microscopy

Epididymal WAT tissues were fixed with 2.5% glutaraldehyde in 0.1 mol/L cacodylate (pH 7.4) and processed as previously described (Briand et al., 2014). Semi-thin sections were prepared (2 μm thick) to validate adipose tissue integrity and were used to measure adipocyte diameters, using Image J software on images captured with an Olympus AX60 microscope equipped with a QIClick Color light camera (QImaging) using QCapture Pro software. Ultra-thin sections were observed with a JEOL JEM-1400 microscope equipped with a digital camera. A minimum of 50-μm membrane stretches, measured at least on 25 different images, has been used for each mouse for caveolae quantification using Image J software.

### Statistical Analysis

Statistical analysis of the results was performed with GraphPad Prism 6.0 (GraphPad Software). Adapted statistical tests as indicated in the figure legends were performed and differences were considered significant when *p* value <0.05 and stated as follow: ^∗^ for *p* < 0.05.

## Results

We previously demonstrated that 6 weeks CIH exposure led to insulin resistance in lean mice by durably altering insulin signaling pathway in WAT (Murphy et al., 2017). We performed a CIH exposure protocol (Figure 1A) to investigate whether CIH-induced insulin resistance could be correlated with defects in the expression of caveolin and cavin proteins as well as the structure of caveolae. Hypoxia within the adipose tissue was monitored by the increased expression of REDD1, a hypoxia-inducible protein that has been demonstrated to be involved in metabolic disorders (Regazzetti et al., 2015). REDD1 expression increased five-fold in hypoxic animals when compared to the normoxic group (Figure 1B). Whereas 6 weeks CIH exposure has no significant impact on mice total body weight nor on adiposity or adipocyte size (Table 1), we confirmed that long-term exposure to CIH increased HOMA-IR (1.05 ± 0.2 and 2.9 ± 0.5, in N and CIH respectively, *p* < 0.05 versus N) showing that CIH induces systemic insulin resistance. To determine whether this systemic insulin resistance could be correlated with altered adipose insulin signaling pathway, we measured the phosphorylation of AKT in response to insulin as a read-out of insulin signaling. We observed that the phosphorylation of AKT on both Thr308 and Ser473 residues in response to insulin is decreased in the CIH group compared to animals exposed to normoxic conditions (Figures 1C,D). As previously shown (Deguchi et al., 2009), hypoxia alone led to a small increase of phosphorylation of AKT on both Thr308 and Ser 473 residues (Figures 1C,D). Therefore, mimicking OSA in mice by exposing animals to 6 weeks of CIH induces systemic insulin resistance and insulin signaling alterations in epididymal WAT.

**TABLE 1:** Baseline characteristics of mice exposed to 6 weeks chronic intermittent hypoxia (CIH) or normoxia (N). g, gram; mg, milligram; μm, micrometer.

	Control	CIH
Mice (*n*)	7	7
Body weight (g)	26.3 ± 0.8	24.9 ± 0.8
Epididymal WAT (mg)	528.3 ± 30.6	603.04 ± 63.8
Epididymal adipocyte diameter (μm)	81.1 ± 4.1	80.2 ± 3.8
Subcutaneous WAT (mg)	348.9 ± 19.2	354.4 ± 27.5
BAT (mg)	82.5 ± 7.8	124.9 ± 12.1
Liver (g)	0.936 ± 0.043	0.984 ± 0.03

**FIGURE 2:**
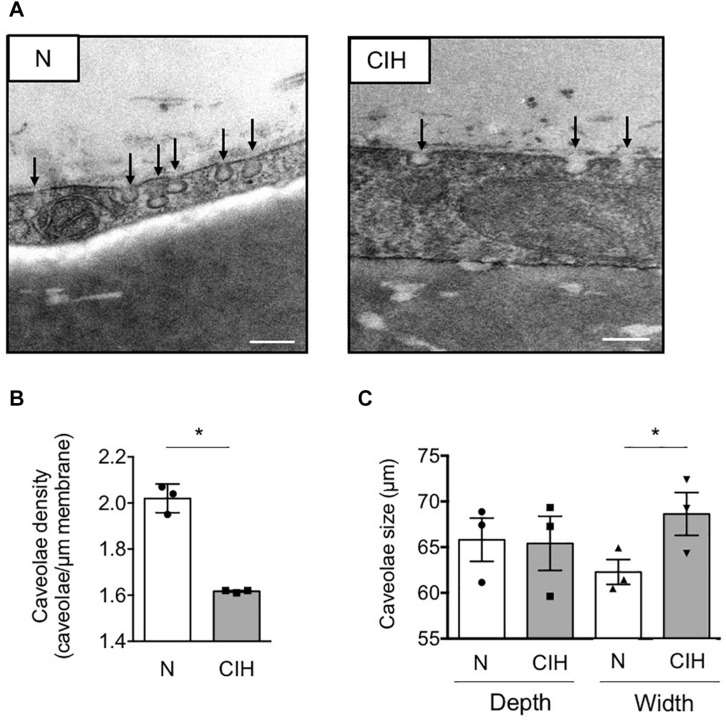
Fat caveolae disassembly along with long-term IH-induced IR in adipose tissue. **(A–C)** Adipose caveolae density is altered by CIH exposure. Electron microscopy images of epididymal WAT following mice exposition to CIH for 6 weeks or normoxia (N) are presented, scale bar: 200 nm **(A)**. Caveolae density is provided for each condition and dot plots represent mean value for each individual animal explored **(B)**. Depth and width of individual caveolae were systematically measured and each dot plots represent mean value for each individual animal explored (*n* = 3 per condition) **(C)**. Data are presented as mean ± sem. vs. normoxic mice (following Student t.test). *p < 0.05, vs. normoxic mice (following Student *t*-test).

In this context of WAT insulin resistance, we next investigated the impact of CIH on caveolae formation by counting the number of flask-shaped caveolae invaginations found in a linear plasma membrane stretch on electron microscopy images (Figure 2A). We observed a significant decrease in membranous adipocyte caveolae density in epididymal WAT from mice exposed to CIH compared to normoxic conditions (Figure 2B). Impact of CIH exposure on caveolae structure was further evaluated by measurement of their depth and width. Although some caveolae structures were still present and maintained on adipocyte plasma membrane in CIH conditions, their size were enlarged in the hypoxic group without affecting their depth, illustrating a tendency of membranous caveolae to flatten (Figure 2C). Thus, exposure of animals to CIH results in flattening of adipocyte membranous caveolae disassembly, concomitant with WAT insulin signaling alterations.

**FIGURE 3:**
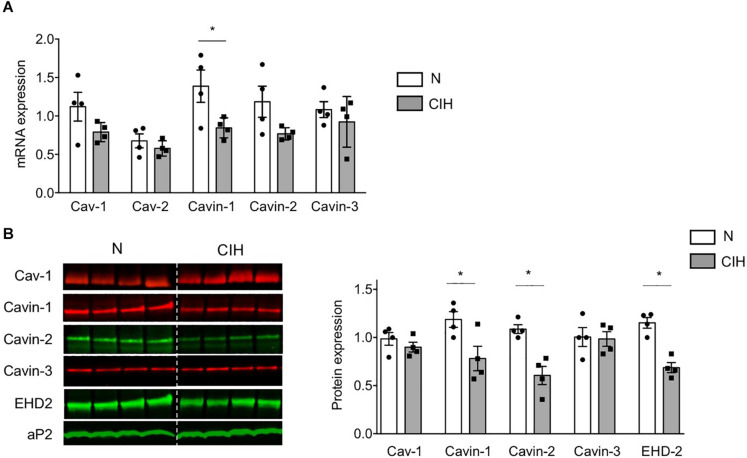
Long-term CIH exposure specifically impacts cavin expression. **(A–C)** mRNA expression of caveolar proteins was determined by quantitative RT-PCR **(A)** and caveolar protein expression by western-blot **(B,C)** in epididymal WAT of mice exposed to Normoxia (N) or CIH for 6 weeks. Quantification of relative mRNA expression of Caveolin-1 (Cav-1), Caveolin-2 (Cav-2), Cavin-1, Cavin-2, and Cavin-3 (normalized to GAPDH mRNA expression) is presented in panel **(A)** (*n* = 4 mice per condition). Immunoblots of caveolar protein expression are presented in panel **(B)**. The dash line separates normoxic (N) and 6 weeks-CIH samples, run on the same blot, but not loaded adjacently. Quantification of caveolin-1/Cav-1, cavin and EHD2 relative protein expressions (normalized to aP2 protein) are presented in panel **(C)**, as mean ± sem with dot plots representing relative protein signal for each mouse explored. Significant differences between conditions was evaluated by Student *t*-test, *p < 0.05 vs. normoxic mice.

In order to gain insights on the mechanisms underlying such caveolae disappearance, we next investigated the expression of adipocyte skeleton protein caveolins and caveolae adaptator proteins cavins, known to regulate caveolae structure (Parton et al., 2020). CIH did not impact Cav-1 and caveolin-2 (Cav-2) mRNA (Figure 3A) nor Cav-1 protein expression (Figures 3B,C). Conversely, cavin-1 mRNA (Figure 3A) and cavin-1 and -2 mRNA protein expression (Figures 3B,C) were significantly altered by prolonged CIH whereas cavin-3 was unaffected (Figures 3A–C). Interestingly, CIH exposition resulted in Eps-15 Homology Domain (EHD) 2 protein decrease (Figures 3B,C). EHD2 is known to stabilize membranous caveolae. The decrease of its expression along the decrease of cavin-1 and cavin-2 expression both traduce caveolae disassembly.

## Discussion

In the present study, we show that mice exposure to CIH results in adipocyte cell membranous caveolae disassembly by downregulating cavin expression, which is concomitant to WAT insulin signaling alterations. This event could be part of the molecular basis of insulin resistance development in OSA patients. Episodes of CIH associated to OSA are recognized as a risk factor for the development of insulin resistance. Nonetheless, molecular mechanisms linking CIH with insulin signaling alterations remain unclear.

As we previously demonstrated for WAT hypoxia related to obesity, CIH-induced caveolae disassembly is accompanied by insulin signaling alterations in WAT and systemic insulin resistance in lean mice (Regazzetti et al., 2015). CIH drives caveolae flattening by downregulating cavin protein expression without any changes in Cav-1 expression. Our results therefore confirm that caveolae structuration is highly dependent on accessory proteins, including cavins and EHD2 proteins, whose release from the plasma membrane have been identified as the signature of caveolae disassembly in many cellular models (Parton et al., 2020). Furthermore, it also demonstrates that Cav-1 expression level does not always reflect membranous caveolae structuration.

We previously reported that extreme adipocyte shrinkage induced by lipolytic stimuli also leads to caveolae disassembly by targeting cavin for degradation (Briand et al., 2014). Activation of lipolysis, which is well-described in response to CIH and associated sympathetic activation (for a review, see Ryan et al., 2019), could also contribute to CIH-induced caveolae disassembly. In our experimental conditions, CIH did not affect mice total body weight nor adiposity or adipocyte size, suggesting that other mechanisms may be involved. A plausible molecular regulation of caveolae structuration may imply HIF-1 transcription factor since we previously demonstrated its ability to downregulate cavin-1 and cavin-2 mRNA expression (Regazzetti et al., 2015; Varela-Guruceaga et al., 2018) in 3T3-L1 adipocytes exposed to hypoxia. Whereas the concomitant induction of HIF-1 target gene REDD1 parallels cavin-1 and 2 mRNA downregulation, further investigations are needed to better investigate a direct regulation of cavin expression by CIH-induced HIF-1 activation. Finally, others demonstrated that CIH-exposed mice develop cardiac dysfunction which is signed by an increase of passive stiffness of myocardial extracellular matrix (ECM) in the heart (Farre et al., 2018). Since caveolae flattening has been identified as a physiological response to buffer membrane tension in response to mechanical stresses (Sinha et al., 2011), one can speculate that CIH could similarly alter WAT ECM and that the resulting mechanical stress may participate in caveolae disassembly process.

Insulin signaling is initiated by the binding of the hormone to the membranous insulin receptor and drives a phosphorylation cascade on downstream insulin signaling proteins. Many insulin signaling mediators, including insulin receptor, are localized in caveolae. Our previous work identified a strong positive correlation between adipose cavin-2 expression and the HOMA-IR of obese diabetic patient (Regazzetti et al., 2015), highlighting the impact of caveolae disassembly in insulin resistance development. A recent study reported that a 3 day-CIH exposure of human coronary artery endothelial cells also impaired insulin-dependent activation of AKT and eNOS, likely through Cav-1 negative eNOS regulation (Sharma et al., 2018). While the authors did not explore caveolae structuration in their cellular model, this recent report together with our data also argues for a role of caveolar protein modulation in response to CIH as a plausible molecular basis to explain insulin-related metabolic dysfunctions.

Altogether, our results strongly suggest that caveolar dysfunction could be a key event in the development of CIH-induced adipose tissue insulin resistance, although we cannot rule out that others mechanisms could be involved. Transcriptional characterization of adipose tissue or adipocyte by single cell genomic analysis could provide important information concerning molecular mechanisms involved. Our observational proof-of-concept study in mice indicates that CIH induces adipose tissue caveolae disassembly and concomitant systemic insulin resistance. Although numerous evidence suggest a central role of HIF-1, mechanistic studies are mandatory to determine specific molecular mechanisms involved in these CIH-induced caveolae disturbances and to demonstrate its subsequent functional consequences. Overall, this study contributes to the understanding of the complex relationship between metabolic disorders and OSA.

## Data Availability Statement

The data, methods used in the analysis, and materials used to conduct the study are available from the corresponding author upon reasonable request. Requests to access the datasets should be directed to soazig.lelay@inserm.fr.

## Ethics Statement

The animal study was reviewed and approved by Cometh Grenoble and MESR (CEEA no. 12).

## Author Contributions

SLL conceived and designed the experiments, researched the data, and wrote the manuscript. CA, SG-P, and EB conceived and designed the experiments, researched the data, and reviewed and edited the manuscript. MV-G, LL, and EA researched the data and reviewed and edited the manuscript. RA reviewed and edited the manuscript. All authors contributed to the article and approved the submitted version.

## Conflict of Interest

The authors declare that the research was conducted in the absence of any commercial or financial relationships that could be construed as a potential conflict of interest.

## Abbreviations

CIH: chronic intermittent hypoxia
ECM: extracellular cellular matrix
EHD-2: Eps-15 homology domain 2
HIF: hypoxia inducible factor
HOMA-IR: HOmeostatic model assessment of insulin resistance
OSA: obstructive sleep apnea
REDD1: regulated in development and DNA damage responses 1
WAT: white adipose tissue.
