# Supplementary material for: Intermittent Hypoxia Mediates Caveolae Disassembly That Parallels Insulin Resistance Development
Source: Front Physiol. 2020 Nov 26;11:565486. doi: 10.3389/fphys.2020.565486 (PMC7726350; doi:10.3389/fphys.2020.565486)

Crude Blots corresponding to protein presented in Figure 1A

Ponceau Red

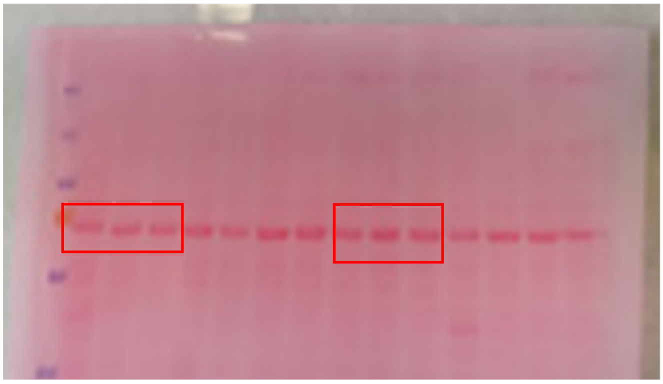

REDD1

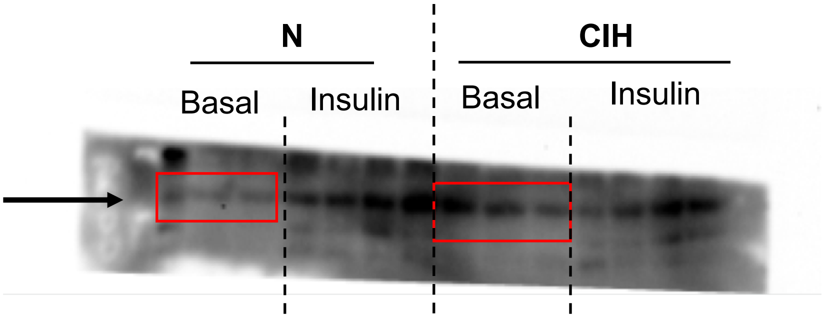

Crude Blots corresponding to protein presented in Figure 1B

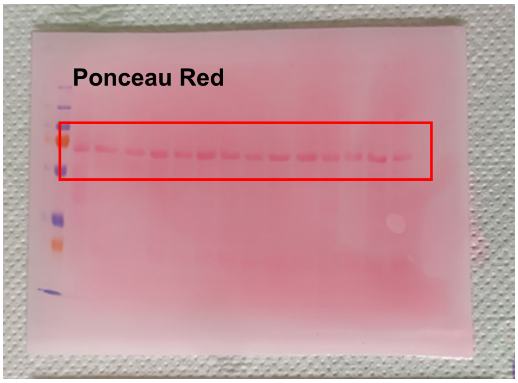

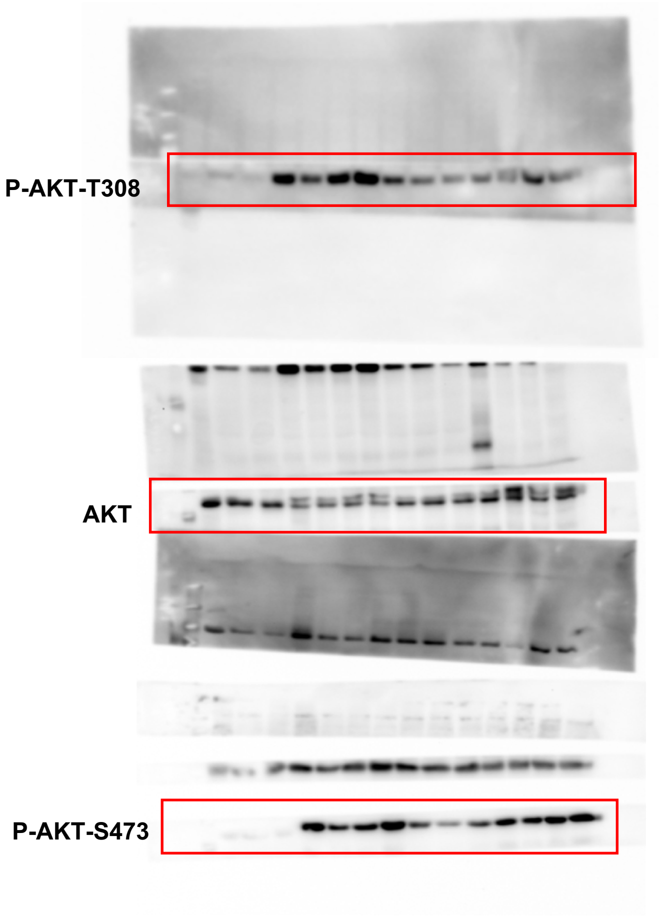

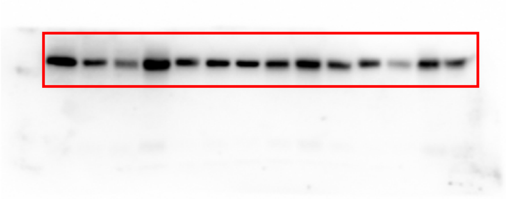

Tubulin

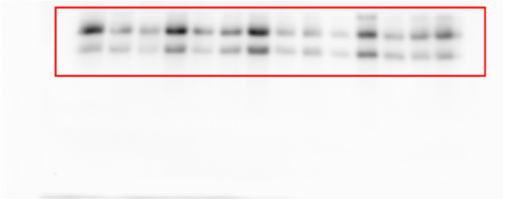

pGSK3

Crude Blots corresponding to protein presented in Figure 3B (1/2)

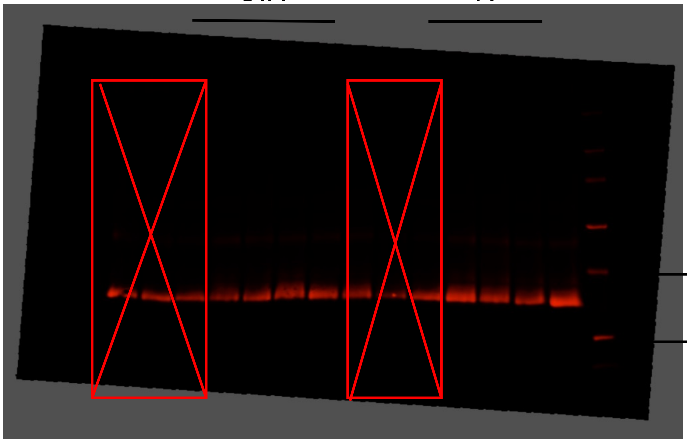

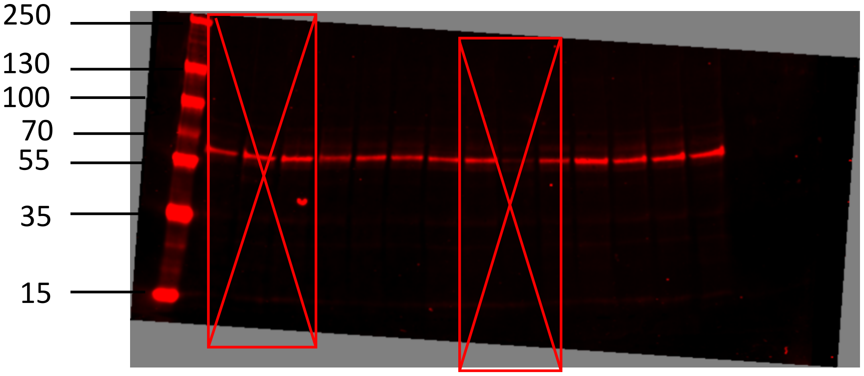

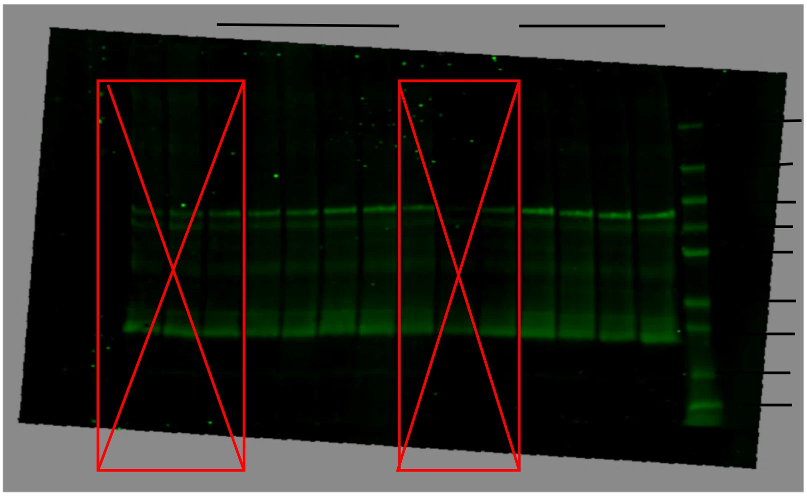

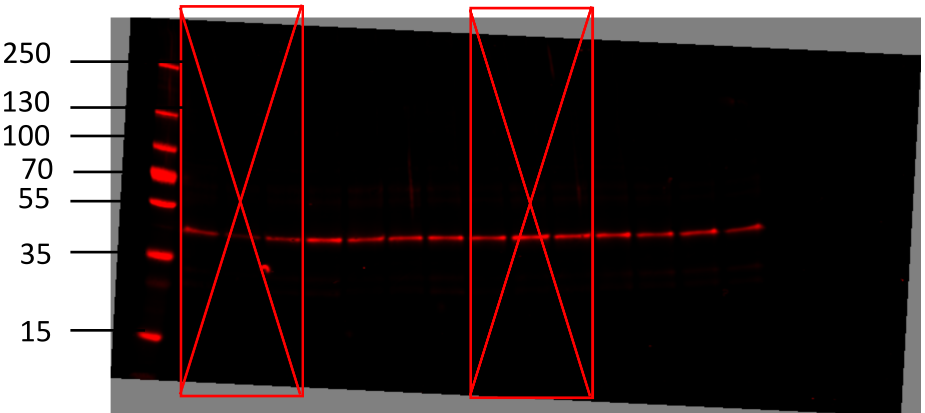

Crude Blots corresponding to protein presented in Figure 3B (2/2)

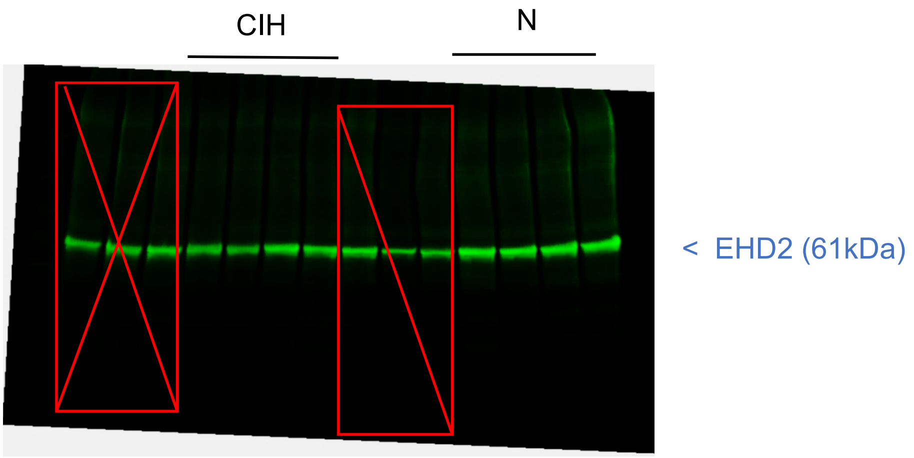

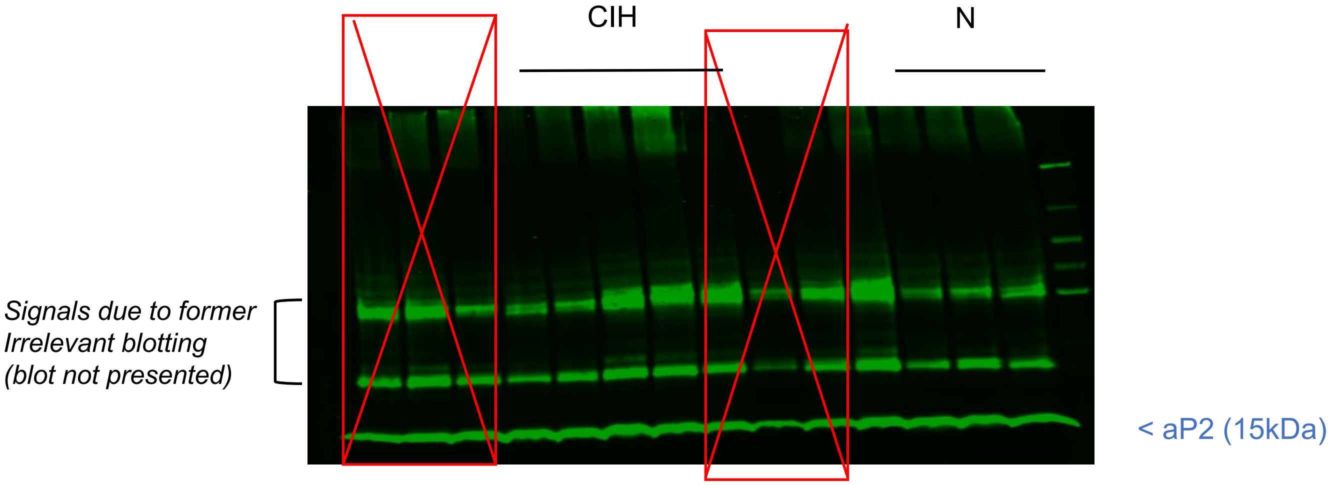

Supplement: Supplementary file 1 [file Data_Sheet_1.PDF]
